# Rapid SARS-CoV-2 Detection Using the Lucira™ Check It COVID-19 Test Kit

**DOI:** 10.3390/diagnostics12081877

**Published:** 2022-08-03

**Authors:** Maya Zahavi, Hanan Rohana, Maya Azrad, Bracha Shinberg, Avi Peretz

**Affiliations:** 1 The Clinical Microbiology Laboratory, Baruch Padeh Medical Center, Azrieli Faculty of Medicine, Bar Ilan University, Poriya, Tiberias 1528001, Israel; mzahavi1@gmail.com (M.Z.); hanan.rohana@gmail.com (H.R.); mazrad@poria.health.gov.il (M.A.); bracha.shainberg@gmail.com (B.S.); 2 Azrieli Faculty of Medicine, Bar Ilan University, Safed 1311502, Israel

**Keywords:** Lucira, COVID-19, LAMP, rapid diagnosis, kit performance

## Abstract

The need for the early identification of SARS-CoV-2 has let to a quest for reliable tests that meet the standards of polymerase chain reaction (PCR) tests, on the one hand, and are low-cost, easy-to-use, and fast, on the other hand. One such test is the Lucira™ Check It COVID-19 Test kit (“Lucira”) (Lucira Health, Inc., Emeryville, CA, USA), which utilizes real-time loop-mediated isothermal amplification technology, developed for at-home use. This study evaluated the clinical sensitivity and specificity of Lucira in identifying the virus in 190 nasopharyngeal samples collected between January and October 2021. Each sample was also subjected to RT-PCR. All negative RT-PCR results were paralleled by a negative Lucira result. Out of 90 participants who had a positive RT-PCR result, 82 (91.1%) tested positive by Lucira. Among the 72 symptomatic participants, 67 (93%) tested positive by Lucira. All samples with a positive RT-PCR result with a threshold cycle (Ct) > 36, yielded a negative Lucira result. In addition, a significant positive correlation was found between Ct and time-to-positivity with Lucira (R = 0.8612, *p* < 0.0001). The implementation of such a portable and affordable assay may aid in breaking the COVID-19 transmission chain.

## 1. Introduction

The severe acute respiratory syndrome coronavirus 2 (SARS-CoV-2), an infection causing the coronavirus disease 2019 (COVID-19), emerged at the end of 2019 in China, and rapidly spread globally, affecting more than 500 million people [1]. Given its high contagiousness, the main strategy of infection control that was immediately pursued was early identification and the isolation of infected individuals. Therefore, the use of an efficient testing method was crucial to blunt the pandemic’s spread. Moreover, an early diagnosis allowed healthcare providers to implement clinically effective intervention for patients at higher risk of developing serious medical complications.

COVID-19 is clinically manifested by various respiratory symptoms such as labored breathing and pneumonia and non-respiratory symptoms such as fever, cough, fatigue, myalgia, headache, and diarrhea [2]. Some patients may be asymptomatic or may present with mild flu-like symptoms [3]. Hence, when considering the use of a diagnostic test for SARS-CoV-2, the effect of symptom variability on test results should be assessed. 

The gold standard method used worldwide for detecting SARS-CoV-2 is the reverse transcriptase-polymerase chain reaction (RT-PCR), a molecular assay with high sensitivity and specificity that identifies the viral RNA in respiratory samples. The different RT-PCR assays mainly target one or several genes, including, for example, the nucleocapsid (N), spike (S), and open reading frame (ORF1ab) genes. However, it requires expensive laboratory instruments and reagents and skilled laboratory personnel and is time-consuming [4]. Just as important, RT-PCR-based assays require high-quality RNA samples [5].

Due to the high magnitude of the pandemic, limited molecular test infrastructure capacities, as well as a shortage of reagents in many countries, there was an eminent need for the development of rapid and easy-to-use tests for SARS-CoV-2 detection [5]. One such assay is the rapid antigen tests, which are based on the identification of specific viral proteins or antibodies against the virus. These tests are fast, cheap, suitable as point-of-care (POC) tests, and do not require highly skilled laboratory technicians. However, most have a lower sensitivity and specificity compared to RT-PCR, especially in samples of asymptomatic patients or patients with low viral loads [6]. Another type of test developed for rapid SARS-CoV-2 detection is based on reverse transcriptase loop-mediated isothermal amplification (RT-LAMP). Although antigen tests are cheaper, the RT-LAMP is more accurate [7]. The novelty of this technique lies in the performance of a PCR reaction at a constant temperature by the use of specific DNA polymerase. This enables rapid and efficient amplification without need for a thermal cycler. 

The LAMP technique is highly specific as it can recognize up to six distinct regions on the viral RNA [8]. The reaction involves two steps: the starting structure-producing step and the cycling amplification step. The starting structure-producing step requires the use of four specially designed target-specific primers (inner primers: FIP and BIP and outer primers: F3, and B3). The Bst DNA polymerase initiates DNA synthesis from FIP and then the F3 primer connects to the target DNA, and the newly synthesized strand displaces the target DNA, which is released. The 3′ end of the released strand then forms a loop structure. Next, a new strand is synthesized using the BIP and B3 primer in the same manner as FIP and F3 and, again, a loop structure is formed, creating a dumbbell-like structure. Thereafter, the amplification stage begins with the conversion of the dumbbell-like structure to stem–loop DNA by self-primed DNA synthesis and proceeds with strand displacement DNA synthesis so that a complement of the dumbbell-like structure is formed. Further elongation and recycling strep reactions yield the desired elongated products [9]. 

One such an assay is the new Lucira™ Check It COVID-19 Test kit (“Lucira”) (Lucira Health, Inc., California, USA), which can detect SARS-CoV-2 from a few copies of RNA in less than 30 min. Based on the clinical trials performed by the manufacturer comparing it to laboratory PCR methods, the kit achieved 98% accuracy [10]. The kit has Emergency Use Authorization (EUA) from the FDA for either home-use by individuals aged 14 and older or for point-of-care (POC) use for all ages by a healthcare provider [11]. Among the three molecular tests that have an EUA authorization and are commercially available, the Lucira kit and the Detect Start Kit cost USD 75, while the Cue is much more expensive; although the tests are sold in 3 or 10 test packs, which may reduce the test cost, only the Cue Reader costs USD 249. While these POC tests are mostly cheaper than a PCR test, their price is much higher (3–10-fold) than an antigen test. However, it should be remembered that antigen tests are less sensitive than molecular-based assays [12]. 

In the current study, we evaluated the clinical sensitivity and specificity of Lucira by comparing its results to those of RT-PCR with samples of both symptomatic and asymptomatic patients. To best of our knowledge, this is the first assessment of the Lucira’s performance. 

## 2. Materials and Methods

### 2.1. Study Population

The study population included 190 participants aged ≥ 18 years who were tested for SARS-CoV-2 between January and October 2021 at the Baruch Padeh Medical Center, Poriya, Israel. Each subject signed a consent form before enrollment. All experiments were performed according to protocols and guidelines approved by the institutional review board of Poriya-Padeh Medical Center in Israel (0066-21-POR).

### 2.2. Detection of SARS-CoV-2 RNA by RT-PCR

Nasopharyngeal samples were collected by trained personnel using flexible nylon flocked swabs (Lingen Precision Medical Products (Shanghai) Cp. Ltd., Shanghai, China) into sampling tubes containing 2 mL virus transport medium (VTM). First, 200 µL samples were mixed with 150 µL lysis buffer (Backman Coulter, Indianapolis, IN, USA) and incubated for 30 min at room temperature. RNA extraction was performed using a Biomek i7 Automated Workstation (Backman Coulter, Indianapolis, IN, USA) in accordance with the manufacturer’s instructions. Then, RT-PCR was performed using the TaqPath RT-PCR COVID-19 Kit (Applied Biosystems™, Thermo Fisher Scientific, Waltham, MA, USA) with a Quanstudio5 Detection System (Applied Biosystems™, Thermo Fisher Scientific, Waltham, MA, USA). A positive result was determined by the threshold cycle (Ct) values (range of 0–40) of the N, S, and ORF1ab genes, as per the RT-PCR’s kit instructions and the Israeli Ministry of Health guidelines. 

### 2.3. Detection of SARS-CoV-2 RNA by the Lucira™ Check It COVID-19 Test Kit

**Figure 1:**
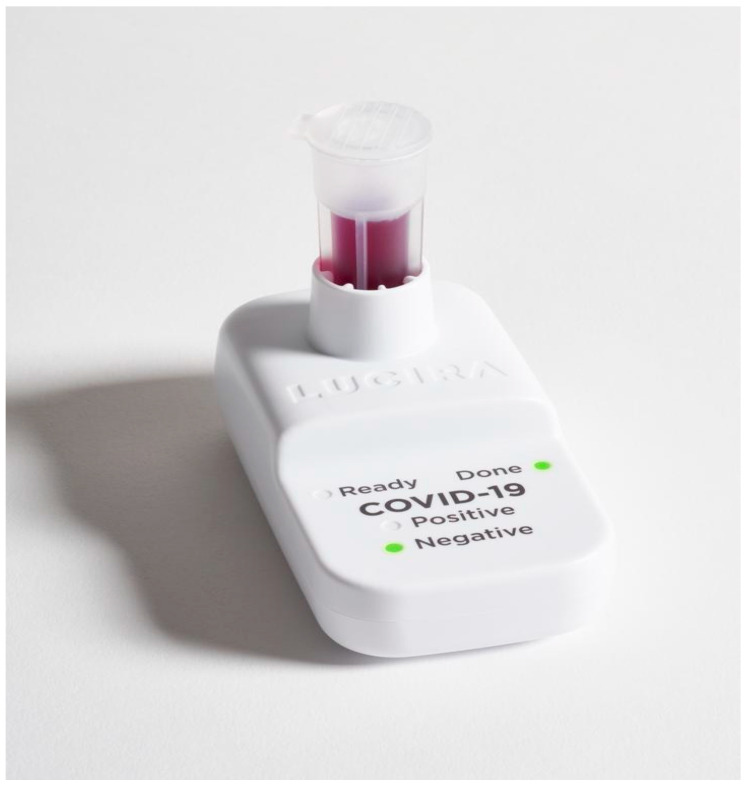
Lucira™ Check It COVID-19 Test kit.

Samples were collected using a nasal swab (provided with the kit), which was rotated 5 times around the internal wall of both nostrils. The swab was then inserted into a sample vial provided with the kit until it touched the bottom, and the swab was then stirred in the provided solution 15 times. The swab was discarded and the vial was then pushed down into the test unit until it clicked (Figure 1). A “Ready” light bulb starts blinking when the test starts running. The “Done” and “Test Result” light bulbs light up when the test is ready (≤30 min). When all lights flash simultaneously, the test is invalid.

Time-to-positivity was measured as the time from the insertion of the vial into the test unit until the “Test Result” light bulb started to blink.

### 2.4. Statistical Analysis

The RT-PCR was set as the reference method for calculating clinical sensitivity and specificity. Therefore, specimens that were found to be positive or negative by the RT-PCR were defined as “True Positive” or “True Negative”, respectively.

Spearman correlation coefficient was calculated to determine the association between the Ct value of RT-PCR to time-to-positivity of the Lucira.

The non-parametric Mann–Whitney test was used to analyze the difference in the distribution of Ct values (RT-PCR) between patients with RT-PCR^+^/Lucira− results and patients with RT-PCR^+^/Lucira+ results.

All tests applied were two-tailed, and a p value of ≤5% was considered statistically significant. Data were analyzed using the GraphPad Prism version 5.03 for Windows (GraphPad Software, San Diego, CA, USA).

## 3. Results

This study included 190 participants, of whom 100 (52.6%) tested negative for SARS-CoV-2 RNA by RT-PCR and the rest were positive. Among the positive group, 72 (80%) demonstrated some of the clinical manifestations of COVID-19 infection, which included gastrointestinal symptoms, weakness, dry cough, and/or dyspnea.

All participants who tested negative by RT-PCR tested negative by Lucira as well. Out of the 90 participants who tested positive by RT-PCR, 82 (91.1%) had a positive Lucira result. Out of the 72 RT-PCR-positive participants who were symptomatic, 67 (93%) tested positive by Lucira. The results are summarized in Table 1. Taken together, Lucira’s clinical sensitivity was 91.1% (CI: 83.23–96.08) while its clinical specificity was 100% (CI: 96.38–100). Among the symptomatic patients, Lucira’s clinical sensitivity was 93.06% (CI: 84.53–97.71).

**Figure 2:**
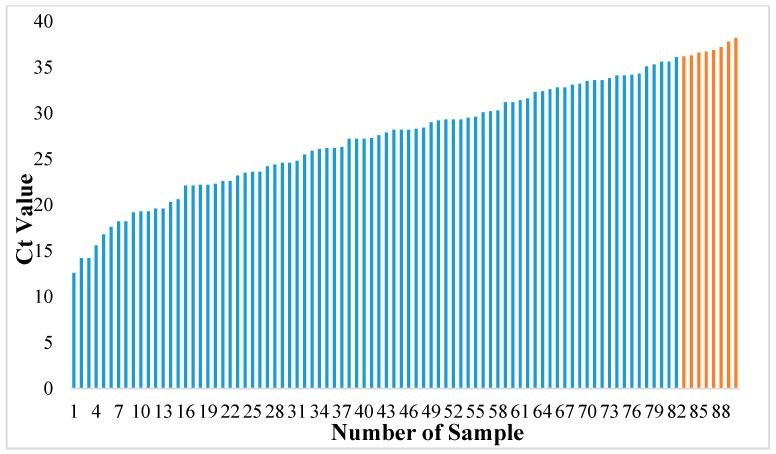
Distribution of Ct values of RT-PCR positive results. Blue bars represent samples of patients for which both the Lucira kit and RT-PCR were positive. Orange bars represent patient samples for which the Lucira kit gave a negative result (Ct value > 36).

**Figure 3:**
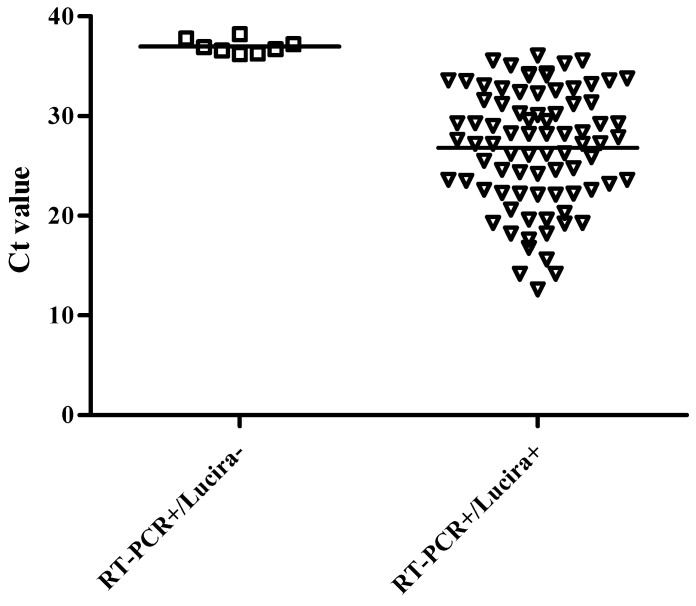
Comparison of Ct values of samples with RT-PCR-positive results between patients with a RT-PCR+/Lucira− results and patients with PCR+/Lucira+ results. The lines represent the mean Ct value.

All patient samples with a Ct value > 36 yielded a negative Lucira result. All eight false negative samples by the Lucira had a Ct > 36 (Figure 2). These eight samples with had a mean Ct value of 37, which was significantly higher than the mean Ct of samples of patients with RT-PCR+/Lucira+ results (26.8) (*p* < 0.001) (Figure 3).

**Figure 4:**
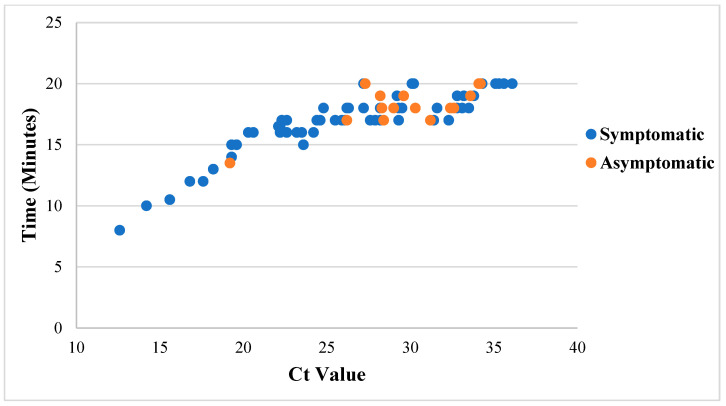
Distribution of Ct values (RT-PCR) against time-to-positivity (Lucira). The blue dots represent samples of symptomatic patients and the orange dots represent samples of asymptomatic patients.

Analysis of time-to-positivity (in the Lucira) of samples from symptomatic and asymptomatic patients found that the average time-to-positivity in samples from symptomatic patients was 16.8 min compared to 18.1 min in samples from asymptomatic patients. The lower the Ct value (in RT-PCR), the less time it took for the Lucira to display a positive result (*p* < 0.0001, R = 0.8612 (0.7898–0.9096); Figure 4).

## 4. Discussion

The main study goal was to evaluate the clinical sensitivity and specificity of the Lucira™ Check It COVID-19 Test Kit, which is based on LAMP technology. Kit results were compared to those of the most sensitive RT-PCR tests authorized by the FDA for SARS-CoV-2 detection. The study included symptomatic and asymptomatic patients, as well as COVID-19-negative participants. We found that Lucira accurately identified all negative samples and had no false-positive results. Lucira’s clinical sensitivity was high (91.1%) and slightly higher when considering only symptomatic patients (93.06%). Previous studies have reported on a wide range of sensitivities for LAMP assay (67% to 100%) [13,14,15,16,17,18]; however, some of these studies tested RT-LAMP on extracted RNA and not directly on the original sample [14,15,19], or used a very small sample size [18]. Regarding specificity, most LAMP assays presented with high specificity values (97–100%) [13,15,17,18]. As with most of the other molecular POC SARS-CoV-2 tests that have been authorized by the FDA, the Lucira kit presented good clinical performance. In comparison to another LAMP-based POC test, the “Detect COVID-19 Test” (Detect Inc., Panama City, FL, USA) had a slightly higher positive percent agreement and negative percent agreement compared to the EUA comparator assay and a higher analytical sensitivity [12]. The Talis One COVID-19 test (Talis Biomedical Corporation, Menlo Park, CA, USA), which is also LAMP-based, also has great clinical performance; however, it has not been authorized for self-testing at home [12]. The current analysis also found that all false negative Lucira results were collected from patients whose RT-PCR results had a Ct value > 36, which indicates a low viral load. These findings suggest that Lucira is more accurate in symptomatic patients compared to asymptomatic ones and in patients with a moderate-to-high viral load. Hence, Lucira may be suitable for the identification of individuals who are currently infectious and should be isolated. Our results are supported by previous studies. For example, Yu et al. found that the false negative samples detected by a LAMP-based assay for SARS-CoV-2 detection were all samples with a Ct value above 35 in RT-PCR [20]. Another study demonstrated a correlation between the difference in color change in the RT-LAMP reaction and the Ct value in RT-PCR; the color difference was greater for lower Ct values [17]. Thus, it seems that LAMP assay accuracy, similar to other tests, improves with increased viral load.

A significant correlation was found between Ct values of RT-PCR and time-to-positivity of Lucira results (*p* < 0.0001); in samples of patients whose PCR result had low Ct values, indicating a high viral load, the positive result in Lucira was displayed in a shorter time compared to samples of patients with a Ct value > 36. This finding further supports the conclusion that Lucira performance is better among patients with a moderate-to-high viral load and who are more likely to transmit the infection. In a former study, Kidd and colleagues found a weaker correlation between the Ct values and time-to-positivity (R = 0.431); however, in contrast to the current study, in which we calculated the correlation based on positive samples, Kidd et al. considered all samples, including the RT-LAMP-negative samples [16].

Despite its high accuracy, which has turned it into the gold-standard method for detecting SARS-CoV-2, RT-PCR is time-consuming and expensive. Even the newer PCR-based platforms that do not require RNA extraction still demand dedicated instruments, which are usually not easily portable, and the costs are quite high. Given its high accuracy and ease of operation, the Lucira kit can be used in facilities which require a rapid COVID-19 result such as airports or emergency rooms and intensive care units. The kit has several benefits; first, it was designed for home-use, and thus enables self-testing without the need for specialized equipment or highly trained personnel for results interpretation. Additionally, results are displayed within 30 min. Thus, it can be used as a point-of-care assay. 

A potential disadvantage of the Lucira kit is the possible uneasiness during the nasal sample collection; however, this is common to other SARS-CoV-2 tests as well. Hence, the main disadvantage is the possibility for false negative results. However, as we found that false positives mostly occurred in patients with a low viral load, this inadequacy may not dramatically impact COVID-19 transmission. 

## 5. Conclusions

The Lucira kit is advantageous owing to its high clinical sensitivity (91.1%) and specificity (100%) compared to RT-PCR. Among the symptomatic patients, Lucira’s clinical sensitivity was 93.06.

It provides an accurate test result in a short time. We believe that the implementation of this portable and affordable kit may aid in early detection and contribute to the prevention of the further spread of SARS-CoV-2 infection.

## Figures and Tables

**Table 1:** Comparison of SARS-CoV-2 detection between RT-PCR and Lucira in symptomatic patients and all participants.

	All Participants		Symptomatic Patients	
	N = 190		N = 72	
	**Positive**	**Negative**	**Positive**	**Negative**
RT-PCR	90	100	72	0
Lucira	82	108	67	5
Sensitivity-% (CI)	91.1 (83.23–96.08)		93.06 (84.53–97.71)	
Specificity-% (CI)	100 (96.38–100)			
NPV%	100			
PPV%	92.6 (86.58–96.03)			

## Data Availability

The dataset used and/or analyzed during the current study are available from the corresponding author on reasonable request.
